# Depression in patients with SAPHO syndrome and its relationship with brain activity and connectivity

**DOI:** 10.1186/s13023-017-0658-5

**Published:** 2017-05-25

**Authors:** Jie Lu, Yanping Duan, Zhentao Zuo, Wenrui Xu, Xuewei Zhang, Chen Li, Rong Xue, Hanzhang Lu, Weihong Zhang

**Affiliations:** 1 Department of Radiology, Peking Union Medical College Hospital, Peking Union Medical College and Chinese Academy of Medical Sciences, No1. Shuaifuyuan Street, Dongcheng District, Beijing, China; 2 Department of Psychology, Peking Union Medical College Hospital, Peking Union Medical College and Chinese Academy of Medical Sciences, Beijing, China; 3 State Key Laboratory of Brain and Cognitive Science, Beijing MR Center for Brain Research, Institute of Biophysics, Chinese Academy of Sciences, Beijing, China; 4 Department of Interventional Radiology, China Meitan General Hospital, Beijing, China; 5 Department of Traditional Chinese Medicine, Peking Union Medical College Hospital, Peking Union Medical College and Chinese Academy of Medical Sciences, Beijing, China; 6 Beijing Institute for Brain Disorders, Beijing, China; 7 The Russell H. Morgan Department of Radiology & Radiological Science, Johns Hopkins University School of Medicine, Baltimore, MD USA

**Keywords:** SAPHO syndrome, Depression, Resting state functional magnetic resonance imaging (rs-fMRI), Default mode network (DMN)

## Abstract

**Background:**

Synovitis-acne-pustulosis-hyperostosis-osteitis (SAPHO) syndrome is a rare disease and there is no related literature concerning psychiatric symptoms in SAPHO patients. Thus, we believe that this will be the first paper to explore the episode and the neurobiological basis of depression symptoms in SAPHO patients using resting state functional magnetic resonance imaging (rs-fMRI). Twenty-eight SAPHO patients and fifteen age- and gender- matched normal controls (NC) were consecutively submitted to psychiatric evaluation and rs-fMRI scanning.

**Results:**

46.2% (13/28) of SAPHO patients were diagnosed as depression. The local spontaneous activity study showed that depressed SAPHO (D-SAPHO) patients had decreased amplitude of low-frequency fluctuation (ALFF) in the bilateral ventrolateral prefrontal cortex (VLPFC, attributed to the anatomical structures of Brodmann’s area 47, 45 and 44) and right dorsolateral prefrontal cortex (DLPFC, attributed to the anatomical structures of Brodmann’s area 8, 9 and 46), increased ALFF in the bilateral middle temporal gyrus, when compared to non-depressed SAPHO (ND-SAPHO) patients. The functional connectivity (FC) study disclosed that D-SAPHO patients had an increased FC in the anterior portions of default mode network (DMN) (the bilateral inferior frontal cortex, anterior cingulate cortex and insula cortex), and a decreased FC in the posterior areas of DMN (left middle occipital cortex), when compared to ND-SAPHO patients. Furthermore, correlation analysis revealed that both ALFF and FC values were significantly correlated with depression scores of SAPHO patients.

**Conclusion:**

These results prompt us to understand the underlying pathophysiological mechanism of depression in SAPHO syndrome, and demonstrate that abnormal brain functional areas may serve as effective biological indicators to monitor depression in the future.

**Electronic supplementary material:**

The online version of this article (doi:10.1186/s13023-017-0658-5) contains supplementary material, which is available to authorized users.

## Background

Synovitis-acne-pustulosis-hyperostosis-osteitis (SAPHO) syndrome is a special kind of clinical entity that characteristically affects the bones, joints, and skin [1]. Inflammatory osteitis with hyperostosis is the main feature of this disease and may occur without skin lesions. SAHPO syndrome is a rare disease and its prevalence is generally considered less than 1/10,000, although sufficient data on it is unavailable [2]. So far, the etiology, pathophysiological mechanisms, treatment and long-term prognosis of SAPHO syndrome have not been fully understood. Most of researchers only focus on dermatological and osteoarticular changes of SAPHO patients. Furthermore, there is no related literature concerning about depressive symptoms in SAPHO syndrome.

Resting state functional magnetic resonance imaging (rs-fMRI) can reveal intrinsic functional architecture of the brain by measuring the spontaneous fluctuations in blood oxygenation level dependent (BOLD) signals in brain during resting state [3]. Amplitude of low-frequency fluctuation (ALFF) and functional connectivity (FC) are two common rs-fMRI data analysis methods. The ALFF reflects the extent of spontaneous neuronal activity [4], while the FC reveals the tendency of cortical networks to be co-activated [5]. Both methods have been applied effectively to detect the mechanisms of pathophysiology of major depressive disorder (MDD)[6–12] and other mental disorders, such as autism spectrum disorders[13], schizophrenia[14], obsessive-compulsive disorders[15] and so on. One of the most widely studied resting state networks is the default mode network (DMN), which plays an important role in self-referential, emotional processes, episodic memory and perceptual processing [16]. A great variety of abnormal regions have been revealed in MDD, mainly including the prefrontal cortex, anterior cingulate cortex, cerebellum, amygdala and so on [17, 18].

Spondyloarthritis (SpA), a family member of immune-mediated inflammatory disorders which includes ankylosing spondylitis (AS), psoriatic arthritis (PsA), reactive arthritis, and undifferentiated SpA, is considered to be associated with depression [19], and SAPHO syndrome may be one subtype of SpA. Baysal et al. [20] reported that the depression had interaction with disease activity and quality of life in AS patients. A recent population-based study revealed that MDD increased the risk of developing PsA among psoriasis patients. Therefore, it is important to identify depression in SAPHO patients as it may have similar effect on AS/psoriatic patients. Our research team is involved in the largest cohort study of SAPHO syndrome in the world [21]. When assessing the clinical, laboratory and radiological features of SAPHO syndrome, we also tried to explore the episode of depressive symptoms in SAPHO patients and revealed neurobiological basis of depression symptoms in these patients using rs-fMRI.

## Methods

### Subjects

Twenty-eight SAPHO patients (aged 16–65, mean 44.6 years, 15 females) were consecutively admitted from inpatient clinics in Peking Union Medical College Hospital from July 25, 2015 to October 20, 2015. All of the SAPHO patients met the diagnostic criteria proposed by Kahn and Khan [1] and had typical anterior chest wall and dermatological manifestations, detailed data shown in Additional file 1: Table S1. There are no uniform scoring criteria to evaluate the severity of SAPHO syndrome, and both SAPHO syndrome and ankylosing spondylitis (AS) are considered to belong to SpA. Therefore, we use the scoring criteria of AS to describe the severity of SAPHO syndrome, including Visual Analogue Scale (VAS) [22], Bath Ankylosing Spondylitis Disease Activity Index (BASDAI) [23] and Bath Ankylosing Spondylitis Functional Index (BASFI) [24]. The drugs that SAPHO patients were using during our study included nonsteroidal anti-inflammatory drugs (NSAIDs), bisphosphonates, and disease-modifying antirheumatic drugs (DMARDs), detailed clinical data of which were shown in Additional file 1: Table S2. Fifteen age- and gender- matched normal controls (NC) (aged 25–65, mean 44.5, 8 females) were enrolled from the local community and they were drug-naïve.

The investigation of depression diagnose was tested by an experienced psychiatrist (Dr.Duan) using the Mini-International Neuropsychiatric Interview (M.I.N.I) [25], and severity of depressive symptoms was tested by the 17-item Hamilton Depression Rating Scale (HDRS) [26]. The group of the depressed SAPHO (D-SAPHO) patients should meet MDD criteria using M.I.N.I. intervention, and HDRS scores should be more than 7. The remaining patients were non-depressed SAPHO (ND-SAPHO) patients. At the same day of psychiatric evaluation, all participants went through MRI scans. The detailed clinical data of all subjects were shown in Table 1.

**Table 1:** Clinical and demographic characteristics

Groups	D-SAPHO (*n* = 13)	ND-SAPHO (*n* = 15)	NC (*n* = 15)	*P* value
	Mean ± SD	Mean ± SD	Mean ± SD	
Ages (years)	49.3 ± 8.7	40.6 ± 13.7	44.5 ± 10.9	0.1642^a^
Gender (M/F)	5/8	8/7	7/8	0.669^b^
Disease duration (months)	37.3 ± 45.1	27.8 ± 42.8	NA	0.5860^c^
HDRS score	18.1 ± 5.3	5.8 ± 2.7	3.1 ± 2.3	<0.0001^a^
VAS	4.0 ± 1.2	4.1 ± 3.4	NA	0.8974^c^
BASDAI	3.6 ± 1.5	2.7 ± 2.3	NA	0.2359^c^
BASFI	2.6 ± 2.4	2.3 ± 2.9	NA	0.9466^c^

D-SAPHO: depressed SAPHO patients; ND-SAPHO: non-depressed SAPHO patients; NC: normal controls; HDRS: Hamilton Depression Rating Scale; VAS: Visual Analogue Scale; BASDAI: Bath Ankylosing Spondylitis Disease Activity Index; BASFI: Bath Ankylosing Spondylitis Functional Index.

^a^The *P* value was obtained by one-way ANOVA.

^b^The *P* value was obtained by a chi-squared test

^c^The *P* value was obtained by two sample *t*-tests

Our study was approved by the local ethic committee of Peking Union Medical College Hospital. The written informed consents before psychometric and neurologic evaluations were signed by all of the subjects.

### MRI Data Acquisition

All subjects were scanned with a 3-Tesla MRI scanner (MAGNETOM Skyra System, Siemens, Erlangen, Germany). Foam pads and the earplugs were used to minimize head motion and acoustic noise. The subjects were asked to stay still with their eyes closed and not think anything particular during the resting state scan. Functional images were collected using an echo-planar imaging sequence (repetition time [TR] =2510 ms, echo time [TE] =30 ms, flip angle = 90°, field of view [FOV] =240 mm × 240 mm, in-plane matrix = 80 × 80, slice thickness/gap = 3/0 mm). Additionally, subjects underwent structural imaging using a T1-weighted magnetization-prepared rapidly acquired gradient-echo sequence (176 slices, TR = 2300 ms, TE = 3.17 ms, TI = 900 ms, flip angle = 8°, FOV = 256 mm × 256 mm, in-plane matrix = 256 × 256, slice thickness/gap = 1/0 mm).

### Rs-fMRI date analysis

The image preprocessing was performed using Data Processing Assistant for Resting-State fMRI (DPARSF) (http://www.restfmri.net) [27], which was based on Statistical Parametric Mapping (http://www.fil.ion.ucl.ac.uk/spm) and Resting-State fMRI Data Analysis Toolkit 1.8 (REST) (http://www.restfmri.net) [28]. The first 10 volumes of the functional images were discarded to allow the magnetization to reach for a steady-state. Slice timing correction, head motion correction and nuisance covariate removal were performed. If the subjects’ head motion was more than 2.5 mm in the X, Y, Z-axis or rotation exceeding 2.5°, the subjects would be excluded. No subject was removed due to head motion in this study. Then functional images were normalized into the space of Montreal Neurological Institute template, using unified segmentation on T1 image, and were resampled to a voxel size of 3 × 3 × 3 mm^3^. Spatial smoothing was used with a 6 mm full-width at half maximum Gaussian smoothing kernel. The data were further processed with the linear detrending and temporally band-pass filtering (0.01–0.08 Hz).

### ALFF analysis

The ALFF analysis was also conducted by using the DPARSF software. The time series of each voxel was first transformed into a frequency domain through using fast Fourier transform and the square root of the power spectrum was obtained. ALFF was calculated as the average over a predefined frequency interval (0.01 Hz to 0.08 Hz), performed on a voxel-by-voxel basis. To reduce the global effects of variability, the ALFF of each voxel was divided by the global mean ALFF value.

### Statistics and ALFF-depression correlation analysis

An analysis of variance (ANOVA) was performed on the ALFF to identify brain areas with significant differences among D-SAPHO patients, ND-SAPHO patients and NC (voxel-level *P* < 0.01, cluster size > 1080 mm^3^/40 voxels, corresponding to a corrected *P* < 0.05 as determined by AlphaSim correction). Then these clusters were extracted as a mask. Two sample t-tests were conducted to compare the ALFF differences among the three groups within the mask (voxel-level *P* < 0.001, cluster size >108 mm^3^ /4 voxels, corresponding to a corrected *P* < 0.05 as determined by AlphaSim correction). The correction thresholds were determined by the Monte Carlo simulations based on the AlphaSim in Analysis of Functional Neuroimage [29], which were implemented on the REST software. To explore the correlation between depression and regional resting state activity, a post-hoc correlation analysis was performed between the HDRS scores and ALFF values which were extracted in regions where significant differences between D-SAPHO patients and ND-SAPHO patients were observed.

### FC analysis

FC analysis was performed to explore the changes of DMN in SAPHO patients. To identify the DMN map, a seed region of interests (ROIs) was placed in the posterior cingulate cortex (PCC; 0X,-53Y, 26Z; seed size of 10 mm × 10 mm × 10 mm). For each subject, the average BOLD time course of voxels within ROIs was plotted. Subsequently, we computed the correlation coefficient between that the BOLD time course of voxels within ROIs and the time course of all the other voxels in the brain. Then, the r-scored maps were converted to z-scores by using Fisher’s r-to-z transformation.

### Statistics and FC-depression correlation analysis

The FC differences among groups were performed by using two sample *t*-tests (voxel-level *P* < 0.001, cluster size > 432 mm^3^/16 voxels, corresponding to a corrected *P* < 0.05 as determined by AlphaSim correction) after the ANOVA analysis. The FC values of these regions showing significant differences in DMN between D-SAPHO patients and ND-SAPHO patients were calculated separately. Subsequently, the correlation of the FC values and HDRS scores were analyzed.

## Results

### Clinical and Demographic characteristics

**Fig 1:**
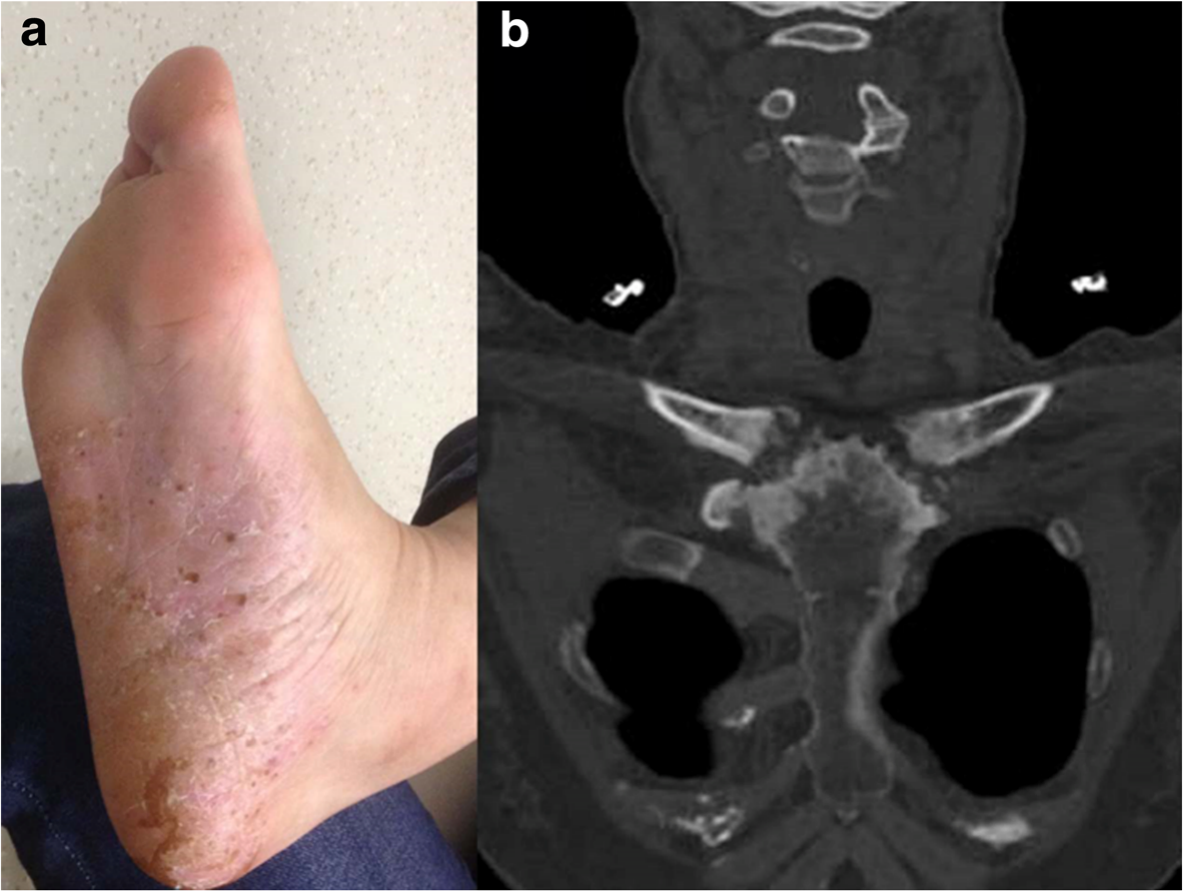
A 50-year-old woman with SAPHO syndrome presented with recurrent palmoplantar pustulosis for seven years and multiple joint pain for six years. (**a**) Erythema with pustules and scales on the right foot. (**b**) Coronal CT image of sternoclavicular joints demonstrates hyperostosis and erosive changes

All of the patients had typical characteristics of syndrome, such as osteoarticular and dermatological lesions, one example shown in Fig. 1, detailed data shown in Additional file 1: Table S1. 46.4% (13/28) of SAPHO patients were diagnosed with depression. There were no significant differences in age (F = 1.891, *P* = 0.1642) and gender (χ^2^ = 0.803, *P* = 0.669) among D-SAPHO patients, ND-SAPHO patients and NC, whereas HDRS scores were significantly different among the three groups (F = 62.82,*P* < 0.0001). There was no significant differences in disease duration (t = 0.5515, *P* = 0.5860), VAS (t = 0.1303, *P* = 0.8974), BASDAI (t = 1.213, *P* = 0.2359) and BASFI (t =0.06762, *P* = 0.9466) between D-SAPHO patients and ND-SAPHO patients, as shown in Table 1. Meanwhile, differences in drug therapies were not significant between the two groups (χ^2^ = 2.610, *P* = 0.302), as shown in Additional file 1: Table S2. There were no significant differences in HDRS scores between male and female patients in either D-SAPHO patients (*t* = 1.852, *P* = 0.0910) or ND-SAPHO patients (*t* = 0.5331*, P* = 0.6030), and the severity of depression was not correlated with age in D-SAPHO patients (*r* = 0.2503, *P* = 0.4095).

### Altered ALFF in SAPHO patients

**Fig. 2:**
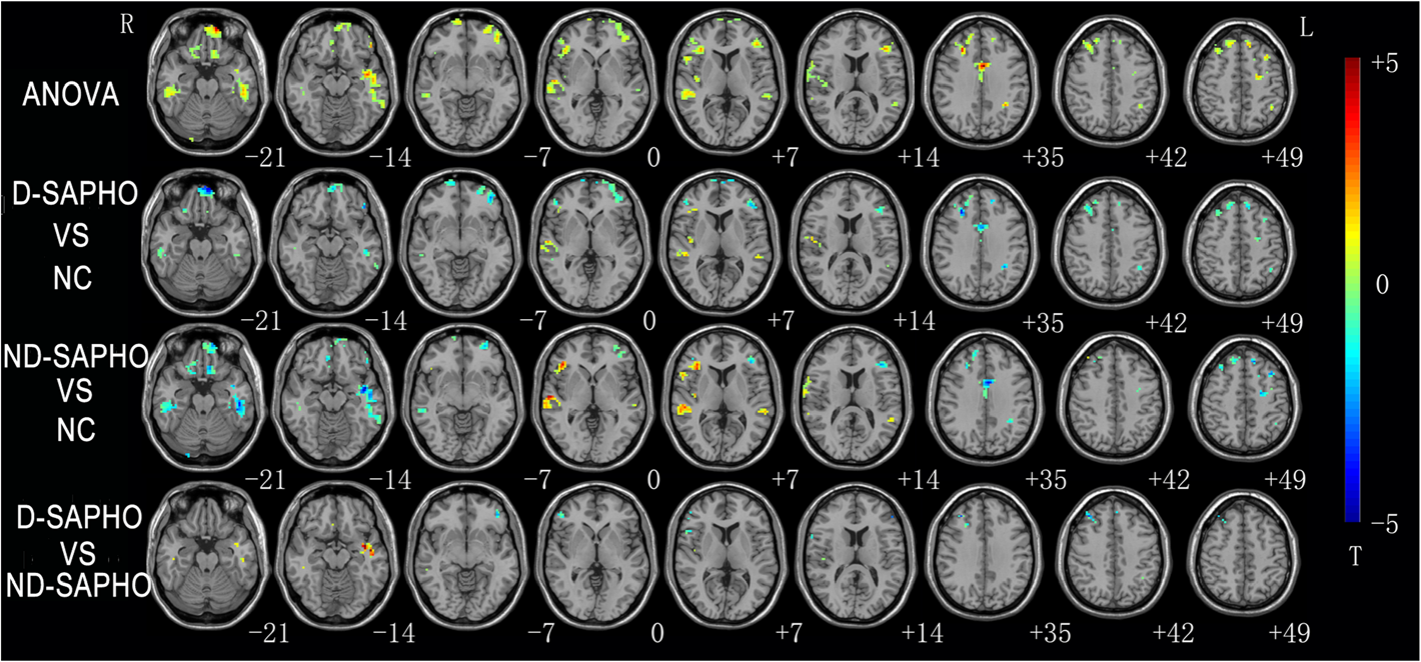
Axial brain slices show the significant differences in the amplitude of low-frequency fluctuation (ALFF) among depressed SAPHO (D-SAPHO) patients, non-depressed SAPHO (ND-SAPHO) patients and normal controls (NC). The color bar represents the range of T values. R = *right*. L = *left*

Significant differences of the ALFF were revealed by ANOVA among the D-SAPHO patients, ND-SAPHO patients and NC in the following regions: bilateral frontal cortex, anterior cingulate cortex, temporal cortex and left inferior partial gyrus (Fig. 2). Then two sample *t*-tests were conducted to compare the ALFF differences among the three groups (Fig. 2): (1) Compared with NC, decreased ALFF was detected in the bilateral prefrontal cortex, inferior temporal gyrus and anterior cingulate cortex in the D-SAPHO patients, and increased ALFF was detected in the bilateral superior temporal gyrus in the D-SAPHO patients. (2) Compared with NC, ND-SAPHO patients demonstrated decreased ALFF in the bilateral prefrontal cortex, anterior cingulate cortex and inferior temporal gyrus and increased ALFF in right superior temporal gyrus, left middle temporal gyrus and right inferior frontal gyrus. (3) Compared with ND-SAPHO patients, D-SAPHO patients showed decreased ALFF in the bilateral ventrolateral prefrontal cortex (VLPFC, attributed to the anatomical structures of Brodmann’s area 47, 45 and 44), right dorsolateral prefrontal cortex (DLPFC, attributed to the anatomical structures of Brodmann’s area 8, 9 and 46), increased ALFF in the bilateral middle temporal gyrus. More detailed information about the group comparisons was shown in Table 2.

**Table 2:** Regions in which ALFFs were significantly different among D-SAPHO patients, ND-SAPHO patients and NC

Brain regions	BA	No. of voxels	Peak MNI coordinates			T value
			x	y	z	
ANOVA results						
Temporal_Inf_R	20	41	60	−21	−33	8.4591
Temporal_Inf_L, Temporal_Mid_L, Temporal Sup_L	20,22	436	−51	−27	−24	23.1744
Temporal_Inf_R	20	117	45	−24	−27	12.4343
Frontal_Sup_Orb_L, Frontal_Mid_Orb_L	11	454	−12	60	−21	25.1148
Rectus_R	11	51	12	33	−27	9.105
Frontal_Sup_Orb_R, Frontal_Med_Orb_R	11	41	12	69	−6	11.4897
Frontal_Inf_Tri_R, Rolandic_Oper_R, Frontal_Inf_Oper_R	45,47,48	237	36	30	6	15.4847
Temporal_Sup_R	22	129	63	−36	9	18.3573
Temporal_Mid_L	21	48	−57	−48	12	11.0862
Anterior_cingulate_cortex_L&R	24	125	−3	6	36	28.988
Frontal_Mid_R	9	121	27	30	36	22.4831
Frontal_Sup_R	9	60	15	36	48	11.4636
Parietal_Inf_L	40	40	−33	−48	36	15.5628
Frontal_Sup_L	6	259	−21	6	66	26.2551
Precentral_L	6	44	−24	−9	45	20.692
Paracentral_Lobule_L	4	79	−6	−36	72	10.9898
D-SAPHO vs NC						
Temporal_Inf_L	20	45	−36	−9	−51	−5.4014
Temporal_Inf_L	20	69	−51	−27	−15	−4.3621
Rectus_L&R	11	110	−3	60	−18	−7.8022
Temporal_Inf_R	20	13	57	−27	−21	−3.4624
Frontal_Inf_Tri_L	45	225	−42	24	18	−5.5312
Frontal_Sup_Orb_R	11	41	12	69	−3	−4.5809
Temporal_Sup_R	22	60	57	−24	3	6.0835
Temporal_Sup_L	22	12	−51	−36	3	3.9613
Rolandic_Oper_R	48	41	54	−12	18	6.089
Frontal_Mid_R	9	101	27	30	36	−6.7464
Anterior_cingulate_cortex_L&R	24	90	−3	6	36	−5.3536
Frontal_Sup_L	6	189	−12	27	54	−5.964
Paracentral_Lobule_L	4	25	−9	−39	75	−4.3756
ND-SAPHO vs NC						
Temporal_Inf_R	20	36	60	−21	−33	−3.5736
Temporal_Inf_L	20	424	−45	−3	−15	−7.2762
Temporal_Inf_R	20	98	36	−21	−27	−4.4523
Frontal_Sup_Orb_L	11	168	−9	24	−21	−4.9596
Rectus_R&L	11	49	6	21	−18	−4.8578
Frontal_Inf_Tri_R	47	156	42	30	0	6.2699
Temporal_Sup_R	22	128	63	−36	9	7.3124
Frontal_Inf_Tri_L	47	80	−42	21	21	−5.3148
Temporal_Mid_L	21	45	−60	−30	3	4.8747
Anterior_Cingulate_Cortex_L&R	24	99	−3	6	36	−6.5394
Frontal_Sup_R	9	17	15	48	36	−4.1507
Precentral_L	6	41	−24	−9	45	−5.6836
Frontal_Sup_R	9	20	15	36	48	−4.0368
Frontal_Sup_L	6	121	−18	6	63	−5.8206
Frontal_Sup_L	6	29	−21	−6	72	−4.4231
Paracentral_Lobule_L	4	46	−15	−21	75	−4.8103
D-SAPHO vs ND-SAPHO						
Temporal_Mid_L	21	54	−39	−6	−12	4.2963
Temporal_Mid_R	21	14	60	−39	−9	3.9518
Frontal_Inf_Orb_L	47	8	−45	42	−3	−3.5221
Frontal_Inf_Tri_R	45	22	51	48	3	−3.556
Frontal_Inf_Oper_R	48	7	51	18	6	−3.2289
Frontal_Inf_Tri_L	46	10	−48	42	18	−4.3255
Frontal_Mid_R	9	28	36	36	45	−3.9717

D-SAPHO: depressed SAPHO patients; ND-SAPHO: non-depressed SAPHO patients; NC: normal controls; BA: Brodmann area; MNI: Montreal neurological institute

**Fig. 3:**
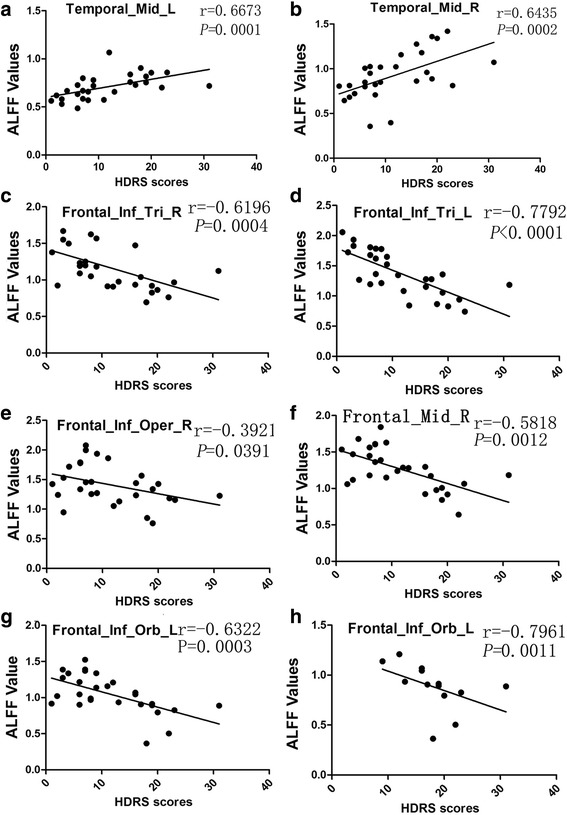
Scatter-plot (**a**-**g**) shows the significant correlation between Hamilton Depression Rating Scale (HDRS) scores of all SAPHO patients (including D-SAPHO patients and ND-SAPHO patients) and ALFF values in regions that show significant differences between D-SAPHO patients and ND-SAPHO patients. Scatter-plot (**h**) shows the significant correlation between HDRS scores of D-SAPHO patients and ALFF values in regions that show significant differences between D-SAPHO patients and ND-SAPHO patients

The relationships between HDRS scores and ALFF in regions showing significant difference (D-SAPHO patients and ND-SAPHO patients) were evaluated. HDRS scores of SAPHO patients (including D-SAPHO patients and ND-SAPHO patients) correlated with ALFF values in all of the regions that showed significant difference between D-SAPHO patents and ND-SAPHO patients (Fig. 3a-g). The left VLPFC (Brodmann’s area 47, Peak: x = −45 y = 42 z = −3) was the only region whose ALFF values correlated with HDRS scores of D-SAPHO patients (*r* = −0.7961, *P* = 0.0011) (Fig. 3h), and the other correlations were not significantly different (*P* > 0.05, no scatter-plot shown).

### Altered FC in D-SAPHO patients

**Fig. 4:**
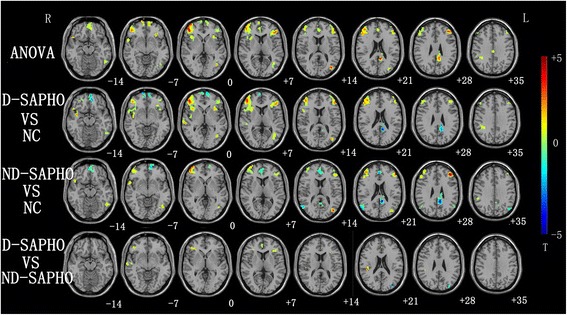
Axial brain slice displays the significant differences in the default mode network (DMN) functional connectivity (FC) among depressed SAPHO (D-SAPHO) patients, non-depressed SAPHO (ND-SAPHO) patients and normal controls (NC). The color bar represents the range of T values. R = *right*. L = *left*

An ANOVA revealed significant differences of the DMN FC among the D-SAPHO patients, ND-SAPHO patients, and NC in the following regions: bilateral prefrontal cortex, bilateral inferior parietal lobe, left posterior cingulate cortex,left inferior temporal cortex, and the right superior pole temporal cortex (Fig. 4). Then two sample t-tests were conducted to determine significant differences of DMN FC among the three group (Fig. 4): (1) The FC results showed that D-SAPHO patients had increased DMN in bilateral prefrontal cortex, inferior parietal cortex, precuneus cortex, left inferior temporal cortex, and middle occipital cortex, while decreased DMN in the bilateral orbital medial frontal cortex and left PCC compared with NC. (2) The results of the FC showed that ND-SAPHO patients had increased DMN FC in the left trigonal inferior frontal cortex, inferior temporal cortex, occipital cortex, inferior parietal cortex, right superior pole temporal cortex, and middle frontal cortex, while decreased DMN FC in the left orbital frontal cortex, PCC, bilateral anterior cingulate cortex, and right inferior parietal cortex compared with NC. (3) Compared with ND-SAPHO patients, D-SAPHO patients showed an increased DMN FC in anterior potions (the bilateral inferior frontal cortex, anterior cingulate cortex and insula cortex), and a decreased DMN FC in posterior areas (left middle occipital cortex). More detailed information about the group comparisons was shown in Table 3.

**Table 3:** Regions in which DMN FCs were significantly different among D-SAPHO patients, ND-SAPHO patients and NC

Brain regions	BA	No. of voxels	Peak MNI coordinates			T values
			x	y	z	
ANOVA						
Occipital_Mid_L, Temporal_Inf_L	19,37	180	−36	−75	12	16.9502
Temporal_Pole_Sup_R	38	46	51	21	−15	10.6324
Frontal_Med_Orb_L	11	188	−9	63	−9	13.376
Frontal_Mid_R,Frontal_Inf_R,Insula_R	45,47,13,9,10,11,29	583	48	45	0	19.9039
Frontal_Mid_L	46	359	−39	39	27	16.233
Cingulum_Ant_R&L	32	86	0	45	21	9.5251
Cingulum_Post_L	31	123	−9	−48	21	17.6861
Parietal_Inf_R	7,40	210	54	−33	48	18.2555
Parietal_Inf_L	7,40	61	−33	−60	48	8.711
D-SAPHO VS NC						
Temporal_Inf_L	37	65	−48	−54	−21	5.5541
Frontal_Mid_Orb_R	11	41	24	45	−21	4.4964
Frontal_Mid_R,Frontal_Inf_R,Insula_R	45,47,13,9,11,29	769	48	45	3	6.2353
Frontal_Med_Orb_L&R	11	173	−6	63	−12	−4.8905
Occipital_Mid_L	19	94	−36	−75	12	5.919
Frontal_Inf_Tri_L,Frontal_Mid_L	45,46	408	−48	39	12	6.0378
Cingulum_Post_L	31	85	−9	−48	21	−5.1808
Parietal_Inf_R	40	291	54	−33	48	6.1014
Precuneus_R	7	88	9	−78	54	4.9475
Precuneus_R&L	5	52	6	−48	57	3.6016
Parietal_Inf_L	7	62	−39	−57	60	3.8855
ND-SAPHO vs NC						
Temporal_Inf_L, Occipital_Inf_L	37,20,19	91	−51	−60	−12	4.3833
Temporal_Pole_Sup_R	38	51	57	15	−9	4.1362
Frontal_Med_Orb_L,Frontal_Sup_Orb_L	11	137	−9	60	−6	−4.6097
Frontal_Mid_R	10,46	354	39	63	3	4.856
Cingulum_Ant_R &L	32	144	3	54	9	−4.7066
Frontal_Inf_Tri_L	46	208	−39	39	27	6.0087
Occipital_Mid_L	19	52	−39	−78	18	5.2247
Cingulum_Post_L	31	179	−9	−45	24	−6.894
Parietal_Inf_R	40	65	−48	−72	39	−4.2049
Parietal_Inf_L	40,7	49	−30	−57	54	4.6654
D-SAPHO vs ND-SAPHO						
Frontal_Inf_Orb_R	47	43	42	36	−3	4.5814
Frontal_Inf_Tri_L, Insula_L	13,29	18	−30	30	6	3.2266
Cingulum_Ant_L&R	32	21	0	42	9	3.301
Rolandic_Oper_R	13	29	45	−33	21	4.2829
Occipital_Mid_L	19	20	−30	−84	24	−4.096

DMN: default mode network; FC: functional connectivity; D-SAPHO: depressed SAPHO; ND-SAPHO: non-depressed SAPHO; BA: Brodmann area; MNI: Montreal neurological institute

**Fig. 5:**
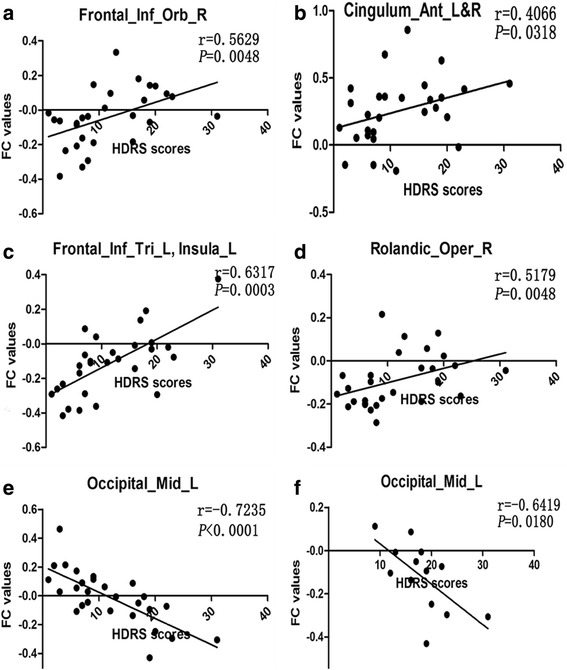
Scatter-plot (**a**-**e**) shows the significant correlation between Hamilton Depression Rating Scale (HDRS) scores of all SAPHO patients (including D-SAPHO patients and ND-SAPHO patients) and the default mode network (DMN) functional connectivity (FC) values in regions that show significant differences between D-SAPHO patients and ND-SAPHO patients. Scatter-plot (**f**) shows the significant correlation between HDRS scores and DMN FC values of D-SAPHO patients in regions that show significant differences between D-SAPHO patients and ND-SAPHO patients

The relationships between HDRS scores and FC in regions showing significant difference (D-SAPHO patients and ND-SAPHO patients) were evaluated. HDRS scores of SAPHO patients (including D-SAPHO patients and ND-SAPHO patients) correlated with FC values in DMN that showed significant difference between D-SAPHO patients and ND-SAPHO patients (Fig. 5a-e). Moreover, the FC between the PCC and left middle occipital cortex (Brodmann’s area 19, Peak: x = −−30 y = −84 z = 24) was significantly correlated with the HDRS scores of D-SAPHO patients (*r* = −0.6419 *P* = 0.0180) (Fig. 5f).

## Discussion

To the best of our knowledge, this is the first study to reveal depressive symptoms in SAPHO syndrome. Our research team previously reported the largest cohort study of SAPHO syndrome in the world, including one hundred and sixty-four patients [21].In this study, psychiatric evaluation and MRI scans were performed on twenty-eight patients of these SAPHO patients, and fourteen SAPHO patients were diagnosed with depression. Therefore, we inferred that the prevalence of depression in SAPHO patients was at least 7.9% (13/164), and much higher than that in Chinese adults (6.40‰) [30]. A study using multivariate logistic regression analysis revealed that severity of disease (BASDAI), quality of life, and educational level were factors associated with the risk of depression in SpA [31]. Thus, we speculated that these factors might also contribute to the episode of depression in patients with SAPHO syndrome.

This study not only confirmed the existence of depression in SAPHO patients by psychiatric tests, but also revealed the abnormal brain activities of D-SAPHO patients by rs-fMRI. We found that D-SAPHO patients showed decreased ALFF in the bilateral VLPFC and right DLPFC, and disrupted FC in DMN.

In our current study, we found decreased local spontaneous activity in the bilateral VLPFC and right DLPFC in D-SAPHO patients compared with both ND-SAPHO patents and NC. As is known, the VLPFC is considered to be negatively correlated with negative affect [32] and inhibitive controls of negative emotions and cognitions, such as reappraisal [33]. Meanwhile, a recent research showed that depression patients had decreased activation of left VLPFC compared to normal controls when processing negative information such as loss [34]. Furthermore, decreased ALFF values in the left VLPFC were negatively correlated to the HDRS score of D-SAPHO patients in our study, which suggested that ALFF measurement in left VLPFC would be a good marker to detect and evaluate the severity of depression in SAPHO patients. The DLPFC has primarily been correlated with cognitive and executive functions and it plays an important role in MDD [35]. Previous studies on MDD patients showed changes of metabolite concentrations in DLPFC [36, 37]. Moreover, a previous study demonstrated that the gray matter density (GMD) of right DLPFC decreased in the MDD patients compared with controls and the GMD values of right DLPFC were negatively correlated with the HDRS scores[38]. In contrast to decreased activity in the bilateral prefrontal cortex, increased ALFF in the bilateral middle temporal gyrus was observed in D-SAPHO patients compared to ND-SAPHO patients. In addition to language comprehension, the temporal lobe also contributes to social cognition and emotional processing [39]. Two previous studies using regional homogeneity (ReHo) analysis [40] and morphometric MRI [41] identified local activity and structure abnormalities in MDD, which were consistent with the pattern of our present findings. Wu et al. [40] demonstrated high ReHo in the right middle temporal gyrus in MDD patients and Ramezani et al. [41] revealed the abnormal structure of medial temporal regions in MDD patients.

Compared to the NC group, decreased ALFFs in the ventromedial prefrontal cortex (VMPFC) were demonstrated in D-SAPHO patients and ND-SAPHO patients separately, nevertheless there was no significant difference in spontaneous activity of VMPFC between D-SAPHO patients and ND-SAPHO patients. As is known, the VMPFC, which is anatomically synonymous with the orbitofrontal cortex, is considered to be involved with the control of emotional, cognitive and social behavior [42]. Firstly, we speculated that rs-fMRI could reveal the abnormal brain activity related to depression of SAPHO patients prior to clinical criteria. In other word, ND-SAPHO patients had the potential to merge depressive symptoms which could be detected by the decreased ALFF in the VMPFC. Secondly, we hypothesized that decreased ALFF in the VMPFC in SAPHO patients could be a SAPHO-specific marker rather than a depression-specific marker.

Further procession by the method of FC in our study, one kind of network level analysis, demonstrated disrupted DMN in SAPHO patients. Moreover, D-SAPHO patients had more FC impairment than ND-SAPHO patients compared to NC. The FC study disclosed that D-SAPHO patients had an increased DMN FC in anterior portions (the bilateral inferior frontal cortex, anterior cingulate cortex and insula cortex), and a decreased DMN FC in posterior areas (left middle occipital cortex), when compared to ND-SAPHO patients. Specially, the FC values between PCC and all of the regions showing significant differences in FC between D-SAPHO patients and ND-SAPHO patients were significantly correlated with the HDRS scores of all SAPHO patients (including D-SAPHO patients and ND-SAPHO patients) in our study, and the FC values between the PCC and left middle occipital cortex was negatively correlated with the HDRS scores of D-SAPHO patients. Our finding indicates that abnormal FCs in DMN are involved in the symptomatology of depression in SAPHO patients. Although we lacked studies exploring the neural mechanisms underlying the functional impairment of DMN in D-SAPHO patients, there are a number of studies in MDD corroborating our results. Similar to our results, Coutinho et al. [43] also described that the FCs of the anterior areas of DMN were positively correlated with depression scores, whereas posterior portions of DMN were negatively correlated with depression scores. These results could be interpreted as dissociation between anterior and posterior FCs in DMN, in which anterior regions could be involved in self-referential and emotional processes and posterior potions could be involved in episodic memory and perceptual processing [16]. A previous review found that patients with MDD exhibited changed connectivity between the anterior DMN and posterior DMN [44], in consistent to our understanding that disrupted DMN was involved in depression in SAPHO patients.

We recognize some limitations in this study. Firstly, our study was limited by the relatively small SAPHO samples, whereas SAHPO syndrome is a rare disease and previous reports about it usually involved dozens of patients. Secondly, SAPHO patients in our study were not drug-naive, which might lead to a potential confusion on rs-fMRI. It is also worthwhile to mention that differences in drug therapies are not distinguishable between D-SAPHO patients and ND-SAPHO patients in this study.

## Conclusion

In summary, our study demonstrates that SAPHO patients may have the potential to develop depressive symptoms. Abnormal brain functional areas revealed by rs-fMRI with the method of ALFF and FC helped to understand the underlying pathophysiological mechanism of depression in SAPHO syndrome, which may be good biological indicators to monitor depression in the future.

## Additional file

Additional file 1: Table S1. Clinical characteristics of the SAPHO patients. **Table S2.** Drugs which SAPHO patients were using in this study. (DOCX 14 kb)

## Abbreviations

SAPHO: Synovitis-acne-pustulosis-hyperostosis-osteitis
rs-fMRI: Resting state functional magnetic resonance imaging
BOLD: Blood oxygenation level dependent
ALFF: Amplitude of low-frequency fluctuation
FC: Functional connectivity
MDD: Major depressive episode
DMN: The default mode network
SpA: Spondyloarthritis
AS: Ankylosing spondylitis
PsA: Psoriatic arthritis
VAS: Visual Analogue Scale
BASDAI: Bath Ankylosing Spondylitis Disease Activity Index
BASFI: Bath Ankylosing Spondylitis Functional Index
NSAIDs: Nonsteroidal anti-inflammatory drugs
DMARDs: Disease-modifying antirheumatic drugs
NC: Normal controls
M.I.N.I: The Mini-International Neuropsychiatric Interview
HDRS: The 17-item Hamilton Depression Rating Scale
D-SAPHO: Depressed SAPHO
ND-SAPHO: Non-depressed SAPHO
TR: Repetition time
TE: Echo time
FOV: Field of view
DPARSF: Data Processing Assistant for Resting-State fMRI
REST: Resting-State fMRI Data Analysis Toolkit 1.8
ANCOVA: Analysis of covariance
VLPFC: Ventrolateral prefrontal cortex
DLPFC: Dorsolateral prefrontal cortex
GMD: Gray matter density
ReHo: Regional homogeneity
VMPFC: Ventromedial prefrontal cortex
