# Supplementary material for: Depression in patients with SAPHO syndrome and its relationship with brain activity and connectivity
Source: Orphanet J Rare Dis. 2017 May 25;12:103. doi: 10.1186/s13023-017-0658-5 (PMC5445372; doi:10.1186/s13023-017-0658-5)
Supplement: Additional file 1: Table S1. — Clinical characteristics of the SAPHO patients. Table S2. Drugs which SAPHO patients were using in this study. (DOCX 14 kb) [file 13023_2017_658_MOESM1_ESM.docx]

| **S1**  Clinical characteristics of the SAPHO patients | | |
| --- | --- | --- |
| Patients | Skin involvement | Bone involvement |
| D-SAPHO 1 | PPP,PV | ACW, Vertebrae |
| D-SAPHO 2 | PPP | ACW, Vertebrae, Sacroiliac joint |
| D-SAPHO 3 | PPP | ACW, Vertebrae, Sacroiliac joint |
| D-SAPHO 4 | PPP,PV | ACW, Vertebrae |
| D-SAPHO 5 | PPP | ACW, Vertebrae, Peripheral joints |
| D-SAPHO 6 | SA | ACW, Vertebrae, Peripheral joints |
| D-SAPHO 7 | PPP,PV | ACW, Vertebrae |
| D-SAPHO 8 | PPP | ACW, Peripheral joints |
| D-SAPHO 9 | PPP | ACW, Sacroiliac joint |
| D-SAPHO 10 | PPP | ACW, Peripheral joints |
| D-SAPHO 11 | PPP | ACW, Vertebrae, Peripheral joints, Sacroiliac joint |
| D-SAPHO 12 | PPP | ACW, Vertebrae, Peripheral joints |
| D-SAPHO 13 | PPP | ACW, Peripheral joints, Sacroiliac joint |
| ND-SAPHO 1 | PPP | ACW, Vertebrae, Peripheral joints, Sacroiliac joint |
| ND-SAPHO 2 | PPP | ACW, Vertebrae, Peripheral joints |
| ND-SAPHO 3 | PPP | ACW, Vertebrae, Peripheral joints, Sacroiliac joint |
| ND-SAPHO 4 | PPP | ACW, Peripheral joints |
| ND-SAPHO 5 | PPP | ACW, Vertebrae, Peripheral joints, Sacroiliac joint |
| ND-SAPHO 6 | PPP | ACW, Vertebrae, Peripheral joints |
| ND-SAPHO 7 | SA | ACW |
| ND-SAPHO 8 | PPP | ACW, Peripheral joints, Sacroiliac joint |
| ND-SAPHO 9 | PPP | ACW, Vertebrae, Peripheral joints |
| ND-SAPHO 10 | PPP,PV | ACW, Vertebrae, Peripheral joints, Sacroiliac joint |
| ND-SAPHO 11 | PPP | ACW, Vertebrae, Peripheral joints, Sacroiliac joint |
| ND-SAPHO 12 | PPP | ACW, Vertebrae, Peripheral joints, Sacroiliac joint |
| ND-SAPHO 13 | SA | ACW, Peripheral joints |
| ND-SAPHO 14 | PPP,SA | ACW, Vertebrae, Sacroiliac joint |
| ND-SAPHO 15 | PPP | ACW, Vertebrae |
| PPP: Palmoplantar pustulosis; PV: Psoriasis vulgaris; SA: severe acne; ACW: Anterior chest wall.  D-SAPHO patients: Depressed SAPHO patients; ND-SAPHO: Non-depressed SAPHO patients. | | |

**Supplementary tables**

| **S2**  Drugs which SAPHO patients were using in this study. | | | | |
| --- | --- | --- | --- | --- |
|  | NSAIDs | Biphosphonates | NSAIDs+ DMARDs | Total |
| D-SAPHO | 5 | 7 | 1 | 13 |
| ND-SAPHO | 4 | 6 | 5 | 15 |
| Total | 9 | 13 | 6 | 28 |
| NSAIDs: nonsteroidal anti-inflammatory drugs; DMARDs: disease-modifying antirheumatic drugs; D-SAPHO: depressed SAPHO patients; ND-SAPHO: non-depressed SAPHO patients. | | | | |
